# Enhanced trimeric ACE2 exhibits potent prophylactic and therapeutic efficacy against the SARS-CoV-2 Delta and Omicron variants in vivo

**DOI:** 10.1038/s41422-022-00656-4

**Published:** 2022-04-13

**Authors:** Mengjiao Li, Zi-Wei Ye, Kaiming Tang, Liang Guo, Wenwen Bi, Yuyuan Zhang, Yan-dong Tang, Guoguang Rong, Mohamad Sawan, Xin Yin, Ren Sun, Shuofeng Yuan, Bobo Dang

**Affiliations:** 1 Fudan University, Shanghai, China; 2 Key Laboratory of Structural Biology of Zhejiang Province, School of Life Sciences, Westlake University, Hangzhou, Zhejiang China; 3 Center for Infectious Disease Research, Westlake Laboratory of Life Sciences and Biomedicine, Hangzhou, Zhejiang China; 4 Institute of Biology, Westlake Institute for Advanced Study, Hangzhou, Zhejiang China; 5 Department of Microbiology, Li Ka Shing Faculty of Medicine, The University of Hong Kong, Pokfulam, Hong Kong China; 6 State Key Laboratory of Emerging Infectious Diseases, Li Ka Shing Faculty of Medicine, The University of Hong Kong, Pokfulam, Hong Kong China; 7 State Key Laboratory of Veterinary Biotechnology, Harbin Veterinary Research Institute, Chinese Academy of Agricultural Sciences, Harbin, China; 8 CenBRAIN Lab., School of Engineering, Westlake University, Hangzhou, Zhejiang China; 9 CenBRAIN Lab., Westlake Institute for Advanced Study, Hangzhou, Zhejiang China; 10 School of Biomedical Sciences, Li Ka Shing Faculty of Medicine, The University of Hong Kong, Pokfulam, Hong Kong China

**Keywords:** Molecular biology, Mechanisms of disease

Dear Editor,

The outbreak of coronavirus disease 2019 (COVID-19) has resulted in a severe global pandemic that has lasted for more than two years.^1^ Several vaccines were developed at an unprecedented speed to protect billions of people.^2^ Many monoclonal antibodies have also been discovered, several of which are in the clinical stages.^3^ However, limited vaccine availability and vaccine hesitancy compromise the impact of vaccines. The continuous evolution of SARS-CoV-2 has also resulted in many variants that can escape immunity, reducing the effects of vaccines and antibodies.^4^ With more than 30 mutations in the spike protein, the Omicron variant escapes the majority of existing antibodies and extensively evades vaccine-induced immunity.^5,6^ Thus, therapeutics that are broadly effective against current and future emerging SARS-CoV-2 variants are still highly desirable.

We and others have envisioned that an engineered ACE2 decoy protein would have the broadest neutralizing activity, which could overcome the viral mutational escape problem.^7–11^ We recently reported that the trimeric ACE2 protein (T-ACE2) could maximally enhance the binding affinity of spike protein to neutralize SARS-CoV-2.^9^ Herein, we further engineered T-ACE2 to improve its neutralizing activity to generate one of the strongest entry inhibitors and demonstrated that this enhanced T-ACE2 (eT-ACE2) protein possessed potent prophylactic and therapeutic efficacy against the Delta and Omicron variants in mouse and hamster animal models.

The T-ACE2 protein comprises three domains: the ACE2 peptidase domain, a linker domain, and a trimerization domain (Fig. 1a).^9^ We first assessed linker domain impact by varying the length of the rigid linker (EAAAK)_n_ and its replacement with rigid (AP)_12_ or (AP)_15_ linkers (Supplementary information, Table S1). (EAAAK)_3_ and (EAAAK)_4_ reduced T-ACE2 neutralizing activity, while the other (EAAAK)_n_ linker constructs showed activities similar to that of T-ACE2 (Supplementary information, Fig. S1). The (AP)_15_ linker construct slightly enhanced the neutralizing activity (Supplementary information, Fig. S1). We then compared the serum stability of (EAAAK)_n_ and (AP)_n_ linkers and discovered that the (AP)_n_ linker is more stable in serum (Supplementary information, Fig. S2). The (AP)_15_ linker protein has greater serum stability than T-ACE2 in vitro (Supplementary information, Fig. S3). Hence, the (AP)_15_ linker construct was chosen for further engineering.

**Fig. 1:**
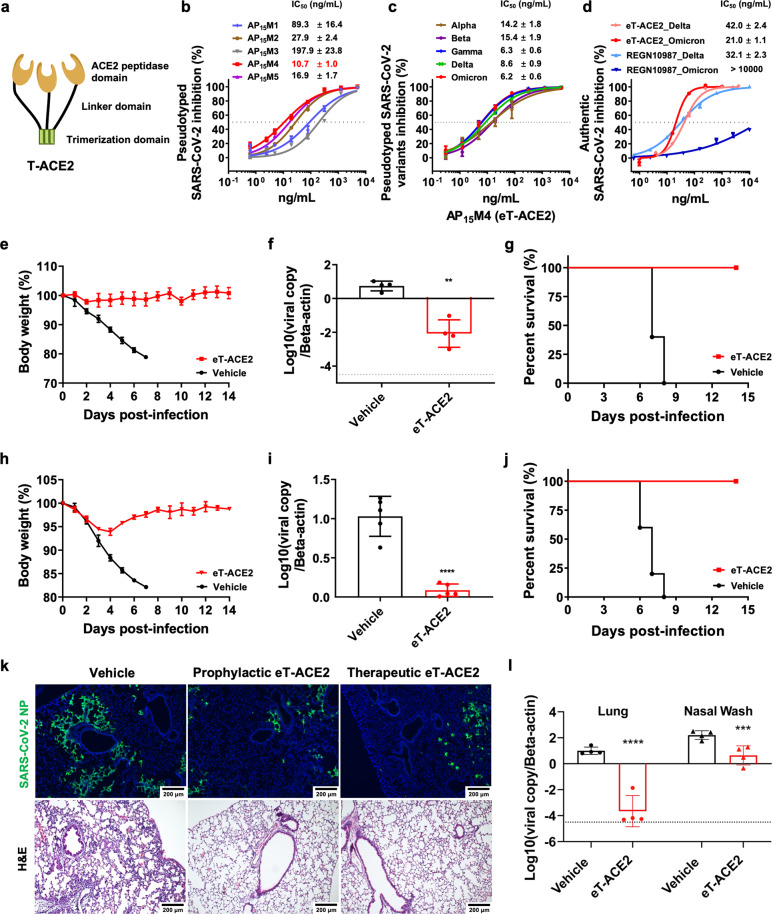
Trimeric ACE2 protein optimization and the inhibition of SARS-CoV-2 variants of concern in vitro and in vivo. **a** The design of the trimeric ACE2 protein (T-ACE2). **b** Inhibitory activities of the trimeric ACE2 proteins incorporating different ACE2 mutations. **c** AP_15_M4 inhibition of pseudotyped SARS-CoV-2 variants of concern. **d** Optimized eT-ACE2 and REGN10987 inhibition of authentic Delta and Omicron variants in microneutralization assays. **e**–**g** eT-ACE2 prophylactic efficacy study in a mouse model challenged with the Delta variant: **e** body weight documentation (*n* = 5); **f** lung viral load detection at 4 dpi (*n* = 4); **g** survival rate (*n* = 5). **h**–**j** eT-ACE2 therapeutic efficacy study in a mouse model challenged with the Delta variant: **h** body weight documentation (*n* = 5); **i** lung viral load detection at 4 dpi (*n* = 5); **j** survival rate (*n* = 5). **k** Viral NP antigen immunofluorescence staining and lung tissue histological examination. Scale bars, 200 μm. **l** eT-ACE2 prophylactic efficacy study in a hamster model challenged with the Omicron variant; lung and nasal wash viral load detection at 2 dpi (*n* = 4). For **f**, **i**, and **l**, bar graphs represent means ± SD. *P* values were analyzed by unpaired *t*-test, *P ≤ 0.05, **P ≤ 0.01, ***P ≤ 0.001, ****P ≤ 0.0001; dotted line indicates limit of detection.

Modifications on the ACE2 peptidase domain were then assessed, including several mutations that could increase receptor binding domain (RBD) binding affinity (Supplementary information, Table S2).^7,8,10^ Analysis of the ACE2-RBD complex structure revealed that the mutations were unlikely to affect ACE2 binding (Supplementary information, Fig. S4).^12^ To optimize RBD binding for activity improvement, we generated five different trimeric ACE2 constructs (each contains an AP_15_ linker domain) based on these ACE2 mutations (AP_15_M1-AP_15_M5, Supplementary information, Table S2). Pseudovirus assays revealed that the majority of these constructs had enhanced neutralizing activities, with AP_15_M4 being the most active (IC_50_ = 10.7 ng/mL, Fig. 1b). Notably, the activity of AP_15_M4 was 18-fold more potent than that of the parent T-ACE2 protein, and its potency exceeded that of most ACE2 decoy proteins and neutralizing antibodies reported to date. We thus designated AP_15_M4 as eT-ACE2. The neutralizing activity of eT-ACE2 against different SARS-CoV-2 variants of concern (VOCs) was tested, showing that eT-ACE2 could inhibit all these variants with IC_50_ values in the range of 6–15 ng/mL (Fig. 1c). In comparison, most of the neutralizing antibody-based drugs showed strong inhibitory activities towards alpha, beta and delta variants with IC_50_ in the range of 1–100 ng/mL.^5^ However, their inhibitory activities rapidly diminished towards the Omicron variant (IC_50_ > 180 ng/mL).^5^ This demonstrated that eT-ACE2 is significantly more resistant to SARS-CoV-2 mutations compared with antibodies.

We measured eT-ACE2 binding affinity to the spike protein extracellular domain (ECD) and determined that the *K*_D_ value was < 1 pM due to an avidity effect (Supplementary information, Fig. S5). To differentiate the binding affinities of T-ACE2 and eT-ACE2, we measured their binding affinities to RBD proteins. Indeed, we found that eT-ACE2 had significantly higher binding affinities to the RBD proteins than T-ACE2 protein (Supplementary information, Fig. S5).

Next, we evaluated the inhibitory activity of authentic Delta and Omicron variants using a microneutralization assay. The optimized eT-ACE2 protein maintained the potent neutralizing activity (IC_50_ values: 42.0 ng/mL for Delta, 21.0 ng/mL for Omicron, Fig. 1d). REGN10987 inhibition of the Delta and Omicron variants was evaluated as a positive control (Fig. 1d). Similar to most therapeutic antibodies, REGN10987 retained potent inhibitory activity against the Delta variant (IC_50_ = 32 ng/mL), but showed much reduced activity against the Omicron variant (IC_50_ > 10 μg/mL).^6^ To determine the inhibitory activity of eT-ACE2 against different authentic SARS-CoV-2 variants, we conducted a viral load reduction assay with qPCR. Potent inhibition of all authentic SARS-CoV-2 variants (Supplementary information, Fig. S6) corroborated our findings in pseudovirus assays.

We used an established K18-hACE2-transgenic mouse model to examine the prophylactic and therapeutic efficacy of eT-ACE2 (Fig. 1e–k). In the prophylactic efficacy study, we administered eT-ACE2 intranasally (i.n.) at a dose of 1.5 mg/kg (30 μg/mouse). After 6 h, the mice were challenged with 5000 pfu of the Delta variant. At 4 days post-infection (dpi), when the viral loads and histopathological changes were expected to be the most prominent, we found that mice receiving eT-ACE2 treatment had a significantly lower viral load in their lung homogenates than mice in the control group (> 2 log10 reduction, *P* < 0.01, Fig. 1f). All vehicle-treated mice died on or before 8 dpi, whereas eT-ACE2 treatment resulted in full protection (100% vs 0%, *n* = 5) (Fig. 1g). Body weight in the eT-ACE2-treated group steadily increased during the 14-day monitoring period, which indicated sterile infection (Fig. 1e).

Before the therapeutic efficacy study, we measured the plasma concentration of eT-ACE2 after its administration into mice and found that eT-ACE2 could not be efficiently absorbed into the blood through intraperitoneal (i.p.) injection (Supplementary information, Fig. S7). We then administered eT-ACE2 via intravenous (i.v.) injection and discovered a short half-life of only ~1 h. However, due to the potent activity of eT-ACE2, its blood concentration remained at the viral inhibition IC_50_ value 24 h after drug administration (Supplementary information, Fig. S7). To assess drug tolerance, two doses of eT-ACE2 (30 mg/kg) were i.v. injected into mice on two consecutive days. The mice were monitored over one week, and no abnormal behaviors were observed, suggesting that eT-ACE2 was well tolerated.

For the therapeutic efficacy study, we did not expect the eT-ACE2 protein to penetrate lung tissues through i.n. administration, thus administered eT-ACE2 through i.v. injection. The mice infected with the Delta variant (5000 pfu) were i.v. administered 15 mg/kg of eT-ACE2 at 24 and 48 hpi. A slight body weight reduction (~5%) was observed in the eT-ACE2-treated mice from day 0 to day 4, but quickly rebounded after 5 dpi (Fig. 1h). In contrast, the vehicle-treated mice showed a continuous drop in body weight before their death (survival rate: 100% vs 0%, *n* = 5, Fig. 1h, j). Notably, i.v. injection of eT-ACE2 suppressed Delta variant replication by > 1 log10 (*P* < 0.0001, Fig. 1i). This marked decrease in viral replication was also evidenced by a reduction in the viral nucleoprotein (NP) in the lungs detected by immunofluorescence staining (Fig. 1k). The lungs of vehicle-treated mice showed large areas of consolidation and cell infiltrates, whereas eT-ACE2-treated lungs exhibited an improved morphology and very mild infiltrations (Fig. 1k). Taken together, our results suggested that eT-ACE2 conferred protection against SARS-CoV-2 Delta variant challenge in the K18-hACE2 mouse model by reducing viral replication and associated inflammation.

The Omicron variant has recently surged and has been reported to extensively evade immunity.^5,6^ We then explored the prophylactic efficacy of eT-ACE2 against the Omicron variant in an established hamster model. Similar to the protocol for K18-hACE2 mice, i.n. delivery of 1.5 mg/kg of eT-ACE2 was conducted at 6 h before challenging with the Omicron variant (10^5^ pfu). Nasal washes and hamster lungs were harvested at 2 dpi due to the attenuated viral replication of the Omicron variant compared to that of the Delta variant.^13^ Remarkably, the Omicron viral copy number was > 10,000-fold lower in hamster lungs (*P* < 0.0001) and >10-fold lower in nasal washes (*P* < 0.001) after eT-ACE2 treatment (Fig. 1l). These results suggest that prophylactic administration of eT-ACE2 in a hamster model reduces both lung viral load and upper respiratory virus shedding of the Omicron variant. In comparison to the Delta variant, the pathology associated with Omicron is much reduced.^13^ Thus, we did not explore the therapeutic efficacy of eT-ACE2 against the Omicron variant in this hamster model.

An ideal viral entry inhibitor would be resistant to mutational escapes, while maintaining efficacy against current and future variants of SARS-CoV-2. Aiming to develop such an inhibitor, we and others have attempted to engineer the SARS-CoV-2 receptor ACE2 as a decoy protein for viral inhibition. Although viral receptor decoys have never been approved as anti-viral drugs, soluble decoy proteins have been widely used in other indications.^14^ Herein, we optimized T-ACE2 by engineering the eT-ACE2 protein, which greatly boosted the viral inhibitory activity, and potently inhibited all SARS-CoV-2 VOCs tested, including Delta and Omicron; most antibodies lose viral inhibitory activity due to mutational escape. Importantly, we showed that eT-ACE2 exhibited potent prophylactic and therapeutic efficacy against the Delta and Omicron variants in mouse and hamster animal models, which laid the foundation to develop eT-ACE2 or similar proteins for clinical applications.

In addition, the eT-ACE2 protein can be lyophilized for long-term storage without affecting its activity, and can also be stored at 4 °C in solution for weeks without losing activity (Supplementary information, Fig. S8). The superior physical and chemical stability of eT-ACE2 empowers with more potential applications such as SARS-CoV-2 detection and in vitro SARS-CoV-2 inactivation. We thus believe that eT-ACE2 is worthy of further development against SARS-CoV-2 and its variants.

## Supplementary information

Supplementary Information
