# Supplementary material for: Enhanced trimeric ACE2 exhibits potent prophylactic and therapeutic efficacy against the SARS-CoV-2 Delta and Omicron variants in vivo
Source: Cell Res. 2022 Apr 13;32(6):589–92. doi: 10.1038/s41422-022-00656-4 (PMC9007249; doi:10.1038/s41422-022-00656-4)
Supplement: Supplementary file 1 — Supplementary Information [file 41422_2022_656_MOESM1_ESM.pdf]

Supplementary Information

Enhanced Trimeric ACE2 Exhibits Potent Prophylactic and Therapeutic Efficacy against the SARS-CoV-2 Delta and Omicron Variants In Vivo

Materials and Methods

Protein expression and purification

The scaffold of trimeric ACE2 (T-ACE2) constructs has been reported,[1] and we inserted a linker sequence after the ACE2(1-615) sequence, followed by trimerization motifs, an HRV3C cleavage sequence, an eGFP tag and a His8 tag. T-ACE2 was constructed as ACE2(1-615)-$(EAAAK)_5$-Foldon-HRV3C-eGFP-His8 for direct comparison. For different linkers, the linker sequence $(EAAAK)_3$, $(EAAAK)_4$, $(EAAAK)_6$, $(EAAAK)_7$, $(AP)_{12}$, or $(AP)_{15}$ replaced $(EAAAK)_5$ (Table S1). The constructs containing AEC2 mutations were based on the $(AP)_{15}$ linker sequence (Table S2).

The ACE2 (accession number: NM_001371415) peptidase domain (1-615) was cloned from a T-ACE2 plasmid. The genes encoding different linkers and foldon were synthesized by Genewiz (Suzhou, China). All the gene fragments were assembled with a Gibson assembly kit (Cat. C112-01, Vazyme). The assembled fragments were subcloned into pEGFP-N1 for expression. The cloned plasmids were transformed into *Escherichia coli* DH5α for amplification. The amplified plasmids were extracted using a GoldHi EndoFree Plasmid Maxi Kit (Cat. CW2104M, CWBio).

Expi293F cells (Invitrogen) were cultured in SMM 293-TII medium (Sino Biological, Lot. RZ14NO1601) at 37°C under 6% CO_2 in a CRYSTAL shaker (140 rpm). The cells were transiently transfected with trimeric ACE2 protein plasmids and polyethylenimine (PEI) (Polysciences, Cat. 24765-1) when the cell density reached approximately 1.5×10^6/mL. A total of 1.5 mg of plasmid was premixed with 3.9 mg of PEI (1:2.6) in 50 mL of fresh medium for 30 minutes before being added to a 1-L cell culture. The transfected cells were cultured for 84-96 hours before harvesting.

For purification of trimeric ACE2 proteins, cell supernatants were harvested by centrifugation at 1200×g for 5 minutes. Then, the supernatants were loaded onto Ni-NTA beads (Smart-Lifesciences, Cat. SA004100) and washed with washing buffer (10-30 mM imidazole, 1× PBS, pH 7.4). The proteins were then eluted with elution buffer (50 mM imidazole, 1× PBS, pH 7.4). After SDS–PAGE and Coomassie blue staining, the eluents with the highest purity were concentrated to a certain volume for dialysis.

To remove the C-terminal tags of trimeric ACE2 proteins, 15 μg of HRV3C protease (GST-HRV3C-His6, expressed and purified in-house) was added to 1 mg of protein and dialyzed at 4°C overnight. Then, the mixture was loaded onto Ni-NTA beads (Smart-Lifesciences, Cat. SA004100) again to remove the HRV3C protease and C-terminal tags. Further purification was performed by size-exclusion chromatography (Superose 6 Increase 10/300 GL, GE Healthcare) if necessary. SDS–PAGE and Coomassie blue staining confirmed the purity (Fig. S9). Finally, the proteins were concentrated and lyophilized with sterile PBS (pH 7.4) containing 5% trehalose, 5% mannitol and 0.01% Tween-80 for long-term preservation.

Pseudotyped SARS-CoV-2 virus packaging and inhibition

Human primary embryonic kidney cells (293T) and human epithelial cells (Caco-2) were cultured with Dulbecco's modified Eagle's medium (DMEM) containing 10% fetal bovine serum (FBS), 100 mg/mL streptomycin, and 100 U/mL penicillin at 37°C under 5% CO2.

Envelope-encoding plasmids for SARS-CoV-2 spike proteins and a luciferase-expressing vector (pNL4-3.Luc.R-E-) were maintained in-house. Pseudotyped SARS-CoV-2 was generated according to previous studies.[1] Briefly, the envelope-encoding plasmid (20 μg) and pNL4-3.Luc.R-E- (10 μg) were cotransfected into 293T cells cultured in a 10-cm cell culture dish using Vigofect transfection reagent (Vigorous Biotechnology, China) following the manufacturer's instructions. The cells were cultured with FBS-free DMEM for 12 hours, and then the cell culture medium was replaced with fresh DMEM containing 10% FBS. The supernatants containing pseudovirus were harvested 48 hours later, filtered with a 0.45-μm filter (Millipore), stored at -80°C and used for single-cycle infection.

Pseudotyped SARS-CoV-2 inhibition assays were used to evaluate the activities of trimeric ACE2 proteins. In detail, Caco-2 cells were seeded in 96-well cell culture plates at 1×10^4 per well and cultured for 12 hours. All the trimeric ACE2 proteins were diluted with FBS-free DMEM, mixed with pseudotyped viruses (1:1, v/v), incubated at 37°C for 30 minutes, and then added to Caco-2 cells. After 12 hours of infection, the culture medium was replaced with fresh DMEM containing 10% FBS, and the cells were cultured for an additional 36 hours. Then, the cells were lysed with Cell Lysis Buffer

(Promega, Madison, WI, USA), and luciferase activity was detected using a Luciferase Assay System (Promega, Madison, WI, USA). All data were analyzed using GraphPad Prism V8.0 software (GraphPad).

Peptide and protein in vitro serum stability determination

All chemicals were purchased from commercial vendors and used without purification. Fmoc-Rink amide resin were purchased from Hecheng Technology (Tianjing, China). Fmoc-L-Gly-OH, Fmoc-L-Ser(tBu)-OH, Fmoc-L-Glu(OtBu)-OH, Fmoc-L-Ala-OH, Fmoc-L-Lys(Boc)-OH, Fmoc-L-Pro-OH were purchased from GL Biochem. Benzoic acid, [2-[2-(Fmoc-amino) ethoxy] ethoxy] acetic acid (Fmoc-PEG2-OH), N, N-Diisopropyl-carbodiimide (DIC), oxyma, HPLC-grade trifluoroacetic acid (TFA), triisopropylsilane (TIPS) and dithiothreitol (DTT) were purchased from Bidepharm. N, N-dimethylformamide (DMF), dichloromethane (DCM), diethyl ether, HPLC-grade acetonitrile were obtained from Energy Chemical. Solvent A: 0.1% TFA in H_2O, B: 0.08% TFA in acetonitrile.

Peptides P1 (Phenyl-PEG$_2$-GSEAAAKEAAAKGS) and P2 (Phenyl-PEG$_2$-GSAPAPAPAPGS) were synthesized on a 0.1 mmol scale using Microwave Peptide Synthesizer (Biotage, MS-PS002). Benzoic acid or [2-[2-(Fmoc-amino) ethoxy] ethoxy] acetic acid was coupled at 60 °C for 30 min, all other amino acids were coupled at 80 °C for 3 min with 0.5 M Fmoc-protected amino acid, 0.5 M DIC and 0.5 M Oxyma, and deprotected with 20% (v/v) 4-methylpiperidine in DMF. After completion of the stepwise SPPS, the resins were washed thoroughly with DCM and dried under vacuum.

The peptides were then cleaved from the resins and side-chain deprotected by treatment with 2% (v/v) water, 2% (v/v) TIPS and 1% (m/v) DTT in TFA for 3 hours at room temperature (RT). The resulting solution containing peptide was precipitated and washed with cold diethyl ether three times. The obtained gummy-like solid was dissolved in 50% A/B (v/v) and lyophilized.

Fresh blood was obtained from Male BALB/c mice (8 weeks old) from the Laboratory Animal Resources Center of Westlake University. The serum was prepared by centrifugation at 1500 g for 10 min after standing at room temperature for 30 min. Peptide P1 or peptide P2 were individually incubated with fresh serum at 2 mM at 37 °C respectively. Samples were taken at 30 min, 60 min, 240 min and 900 min. Then acetonitrile at 75% final concentration was added to serum samples to precipitate plasma proteins, precipitates were removed by centrifugation at 12000 × g for 5 min. The supernatant was diluted 200 times with 0.1%TFA/H2O (v/v) and analyzed by Agilent 1260-6230 TOF LC/MS.

For protein stability analysis in serum in vitro, T-ACE2 protein or $(AP)_{15}$ linker protein was incubated with serum at 50 ug/mL at 37 °C. Samples were collected at 0, 20 min, 40 min, 60 min, 120 min, 360 min, 480min and 600 min. Protein concentration at each time point was quantified by sandwich ELISA. In-house-prepared WT SARS-CoV-2 spike ECD was the coating agent for a 96-well ELISA plate (Biofil, 190731-080). Following washing with TBST (pH 7.4, 0.05% Tween 20), each well was blocked with 5% nonfat dried milk (Beyotime, P0216) in TBST for 2 hours at RT. Then, diluted proteins or blood plasma samples were added to each well and incubated for 2 hours at

RT. Then, 100 μl of anti-ACE2 antibody (1:5000 dilution, Sino Biological, 10108-T56) was added to each well as the detection antibody for 2 hours at RT, followed by the addition of 100 μl of anti-rabbit antibody (1:5000 dilution, Beyotime, A0208) as the secondary antibody for 2 hours at RT. Between the steps, the plate was washed by filling the wells with 200 μl of TBST. Finally, 100 μl of TMB (Solarbio Life Sciences) was added to each well and incubated for 10-15 minutes, followed by the addition of an equal volume of 2 M HCl to stop the reaction. The optical density was read at 450 nm with a microplate reader (Varioskan LUX). The data were analyzed using GraphPad Prism V8.0, standard curves were plotted using PBS diluted protein samples.

Biolayer interferometry

The interactions of the T-ACE2 or $AP_{15}M4$ (eT-ACE2) proteins with the wild-type (WT), Delta or Omicron spike extracellular domain (ECD)/receptor-binding domain (RBD) were determined using Octet Red96e (Sartorius).

Purified T-ACE2 and $AP_{15}M4$ (eT-ACE2) proteins were biotinylated by using EZ-Link NHS-PEG12-Biotin (Thermo Fisher Scientific, Cat. 21313) following the manufacturer's instructions. Any unreacted biotin was removed by ultrafiltration with an Amicon column (30 KDa MWCO, Millipore, Cat. UFC5010BK).

For kinetic analyses, biotinylated T-ACE2 or $AP_{15}M4$ (eT-ACE2) was captured on streptavidin (SA) biosensors. Biotinylated proteins were diluted to 20 μg/mL in kinetics buffer (PBS with 0.02% Tween 20). The WT SARS-CoV-2 spike ECD (expressed and purified in-house), WT spike RBD protein (expressed and purified in-house), Delta

SARS-CoV-2 spike RBD protein (Sino Biological, 40592-V08H90) or Omicron SARS-CoV-2 spike RBD protein (Sino Biological, 40952-V08H121) were diluted to different concentrations in kinetics buffer.

The sensor baselines were equilibrated in kinetics buffer for 120 seconds. Next, T-ACE2 or $AP_{15}M4$ (eT-ACE2) was loaded until the thickness signal shifted 1.0 nm. After loading, the sensors were washed in kinetics buffer. Then, the sensors were immersed in wells containing S-ECD or RBD for 180 seconds (association phase), followed by immersion in kinetics buffer for an additional 300 seconds (dissociation phase). The background signal was measured using a reference sensor with biotinylated protein but no analytes and was subtracted from the corresponding ACE2 binding sensor. The mean kon, koff, and K_D values were calculated with Octet Data Analysis HT 12.0 software using a 1:1 global fit model, and the theoretical fit with $R^2 > 0.95$. Data were plotted using GraphPad Prism V8.0 software (GraphPad)

In vivo plasma concentration determination using sandwich enzyme-linked immunosorbent assay (ELISA)

Male BALB/c mice (8 weeks old) from the Laboratory Animal Resources Center of Westlake University were used for the protein plasma half-life determination experiment. The mice were adapted to the animal facility for 1 week after quarantine and housed under specific pathogen-free conditions. All animal maintenance and experimental procedures were conducted in accordance with the Institutional Animal Care and Use Committee (IACUC) guidelines of Westlake University (Hangzhou,

China).

Mice were randomly divided into control and experimental groups. After anesthetization with isoflurane (2.5%), the $AP_{15}M4$ (eT-ACE2) protein was injected intraperitoneally into mice at an equimolar concentration (100 μL, 15 mg/kg) or injected intravenously into mice at an equimolar concentration (100 μL, 15 mg/kg). To evaluate the plasma half-life of the $AP_{15}M4$ (eT-ACE2) protein, blood samples were collected by tail bleeding either before injection or at a series of indicated time points after injection. The collected blood samples (in a heparin-coated tube) were centrifuged at 1500 g for 10 min to separate the blood plasma, and a protease inhibitor cocktail (Abcam, ab271306) and 5 mM EDTA were added immediately to the separated plasma, which was then stored at -20°C before further analysis.

The plasma concentration of $AP_{15}M4$ (eT-ACE2) was analyzed by a sandwich ELISA as described above.

Authentic virus inhibition

The SARS-CoV-2 B.1.617.2/Delta (GISAID: EPI_ISL_3221329) and B.1.1.529/Omicron (GISAID accession number EPI_ISL_7138045) strains were isolated from the respiratory tract specimens of laboratory-confirmed coronavirus disease 2019 (COVID-19) patients in Hong Kong.[2] VeroE6-TMPRSS2 cells were maintained in DMEM supplemented with 10% heat-inactivated FBS, 50 U/ml penicillin and 50 μg/ml streptomycin. All experiments involving live SARS-CoV-2 followed the approved standard operating procedures of the biosafety level 3 facility at

the University of Hong Kong, as previously described.[3] REGN10987 (Sotrovimab) was purchased from Trinomab, Zhuhai, China.

Viral load reduction assay

A viral load reduction assay was performed on VeroE6/TMPRSS2 cells as described previously.[4] Supernatant samples from the infected cells (MOI = 0.1) were collected at 48 h.p.i. for qRT–PCR analysis of virus replication. Briefly, 100 μl of viral supernatant was lysed with 400 μl of AVL buffer and then extracted for total RNA with the QIAamp viral RNA mini kit (Qiagen). Real-time one-step qRT–PCR was used for quantitation of SARS-CoV-2 viral load using the QuantiNova Probe RT-PCR kit (Qiagen) with a LightCycler 480 Real-Time PCR System (Roche). Each 20-μl reaction mixture contained 10 μl of 2×QuantiNova Probe RT-PCR Master Mix, 1.2 μl of RNase-free water, 0.2 μl of QuantiNova Probe RT-Mix, 1.6 μl each of 10 μM forward and reverse primer, 0.4 μl of 10 μM probe and 5 μl of extracted RNA as the template. Reactions were incubated at 45 °C for 10 min for reverse transcription, 95 °C for 5 min for denaturation, followed by 45 cycles of 95 °C for 5 s and 55 °C for 30 s. Signal detection was carried out and measurements were made in each cycle after the annealing step. The cycling profile ended with a cooling step at 40 °C for 30 s. The primers and probe sequences were against the RNA-dependent RNA polymerase/helicase (RdRP/Hel) gene region of SARS-Cov-2, as we previously described.[4]

Mouse experiment

Heterogeneous K18-hACE2 C57BL/6J mice were obtained from The Jackson Laboratory. For viral challenge in mice, 6- to 8-week-old female and male K18-hACE2 transgenic mice were anesthetized, followed by intranasal inoculation with the Delta variant of SARS-CoV-2 at 5×10^3 PFU/mouse (20 μl/mouse). For the prophylactic experiment, one dose of $AP_{15}M4$ (eT-ACE2) (1.5 mg/kg) was given intranasally 6 hours before viral challenge. For the therapeutic experiment, two doses of $AP_{15}M4$ (eT-ACE2) (15 mg/kg) were given intravenously at 24 hpi and 48 hpi. The mice were sacrificed at 4 dpi to harvest lung tissues for virological assessment and histological examination, as we previously described.[4] The survival and body weight of the infected animals were monitored for 14 days or until mouse death. Viral load in the lung tissue homogenates was detected by qRT–PCR methods described above. We assigned viral copy detection limit Ct value as 40, whereas Ct value for beta actin is usually 25, thus viral copy/beta-actin = $2^{-(40-25)}$ = 0.0000305, log10(viral copy/beta-actin) = -4.515 and this is the limit of detection.

Hamster experiment

Male and female Syrian hamsters, aged 6-10 weeks old, were kept in biosafety level 3 housing and given access to standard pellet feed and water, as we previously described.[5] All experimental protocols were approved by the Animal Ethics Committee of the HKU (CULATR) and were performed according to the standard operating procedures of the

biosafety level 3 animal facilities. Experimentally, each hamster was intranasally inoculated with 10^5 PFU of SARS-CoV-2 Omicron in 100 μL of PBS under intraperitoneal ketamine (200 mg/kg) and xylazine (10 mg/kg) anesthesia. Six hours before viral challenge, the hamsters were intranasally given one dose of $AP_{15}M4$ (eT-ACE2) or vehicle (1.5 mg/kg) as a prophylactic therapy. The animals were then monitored twice daily for clinical signs of disease. At 2 dpi, four animals in each group were sacrificed, and the viral loads in lung tissue homogenates and nasal washes were determined by qRT–PCR as described above.

Table S1. Different trimeric ACE2 proteins with different linkers.

Proteins #1	Linker Sequences	Number of amino acids
T-ACE2	GS EAAAK EAAAK EAAAK EAAAK EAAAK GS	29 AA
H3	GS EAAAK EAAAK EAAAK GS	19 AA
H4	GS EAAAK EAAAK EAAAK EAAAK GS	24 AA
H6	GS EAAAK EAAAK EAAAK EAAAK EAAAK EAAAK GS	34 AA
H7	GS EAAAK EAAAK EAAAK EAAAK EAAAK EAAAK EAAAK GS	39 AA
$(AP)_{12}$	GS APAPAPAPAPAPAPAPAPAPAPAP GS	28 AA
$(AP)_{15}$	GS APAPAPAPAPAPAPAPAPAPAPAPAPAPAP GS	34 AA

Table S2. Different trimeric ACE2 proteins with different ACE2 mutations.

Proteins #2	ACE2 (18-615) Mutations
$AP_{15}M1$	K31F, H34I, E35Q
$AP_{15}M2$	T27Y, L79Y, N330Y
$AP_{15}M3$	T27Y, H34A
$AP_{15}M4$	T27Y, K31F, H34I, E35Q, L79Y, N330Y
$AP_{15}M5$	T27Y, H34A, L79Y, N330Y

Note. Mutations in $AP_{15}M1$ includes K31F, H34I and E35Q, these mutations were reported to increase RBD binding affinity by 10-fold.[6] Mutations in $AP_{15}M2$ includes T27Y, L79T and N330Y, these mutations were reported to increase RBD binding affinity by 36-fold.[7] Mutations in $AP_{15}M3$ includes T27Y and H34A, these mutations were reported to increase RBD binding affinity by 38-fold.[8] Mutations in $AP_{15}M4$ are the combination of those in $AP_{15}M1$ and $AP_{15}M2$. Mutations in $AP_{15}M5$ are the combination of those in $AP_{15}M2$ and $AP_{15}M3$.

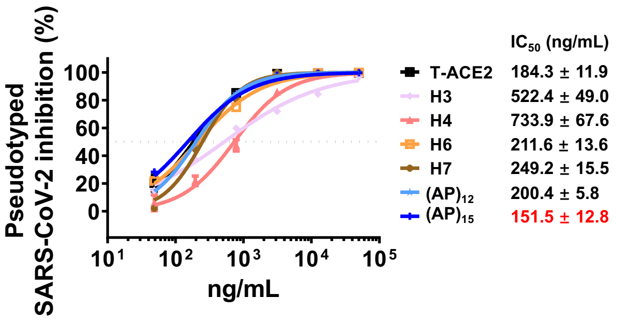

Fig. S1. Linker impact on the inhibitory activity of the trimeric ACE2 proteins. SARS-CoV-2 pseudovirus inhibition assay was employed to assess the inhibitory activities of the trimeric ACE2 proteins with different linkers listed in Table S1, $(AP)_{15}$ linker construct has the best inhibitory activity.

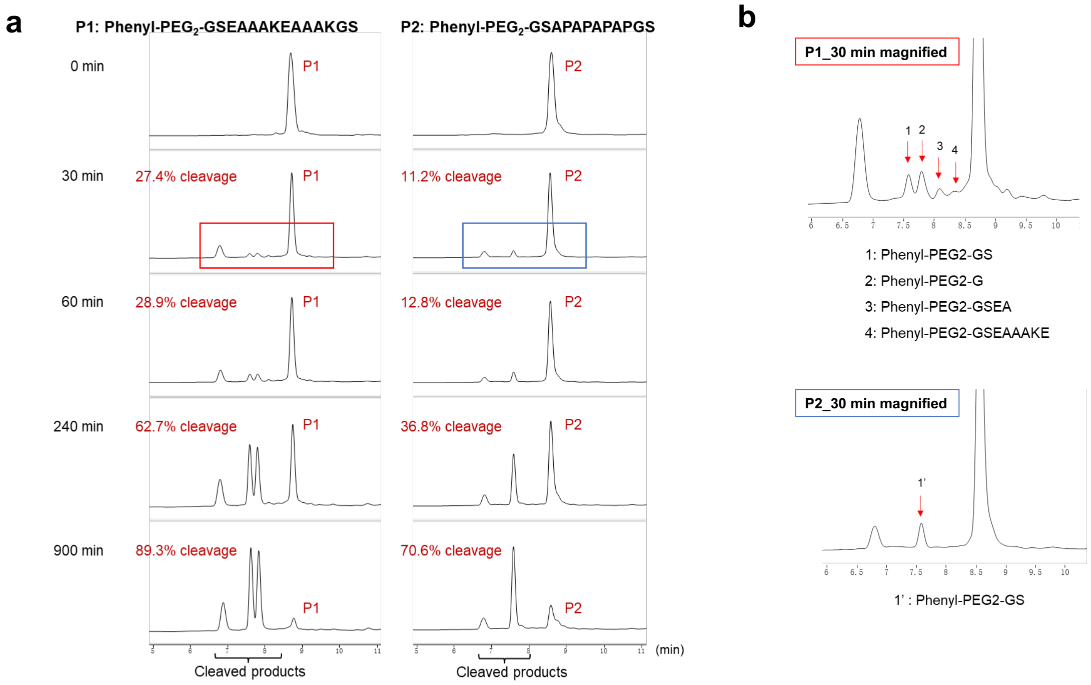

Fig. S2. (EAAAK)n and (AP)n linkers serum stability assessment in vitro. a. Cleavage percentage analysis of the two peptides in serum at different time points. **b.** Different cleaved products analysis when peptides are incubated in serum for 30 min. Note. the peak around 6.5-7 min cannot be identified as peptide cleaved product and was not labeled.

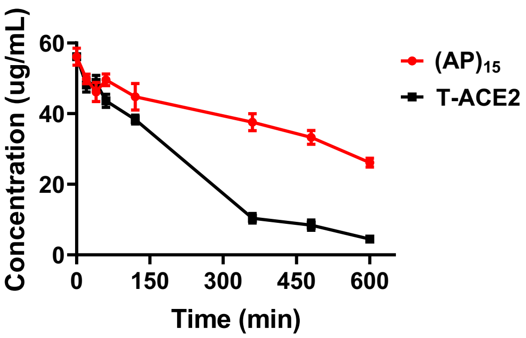

Fig. S3. T-ACE2 and $(AP)_{15}$ linker protein serum stability assessment in vitro. These two proteins were incubated in serum, concentration at different time points were analyzed using sandwich ELISA.

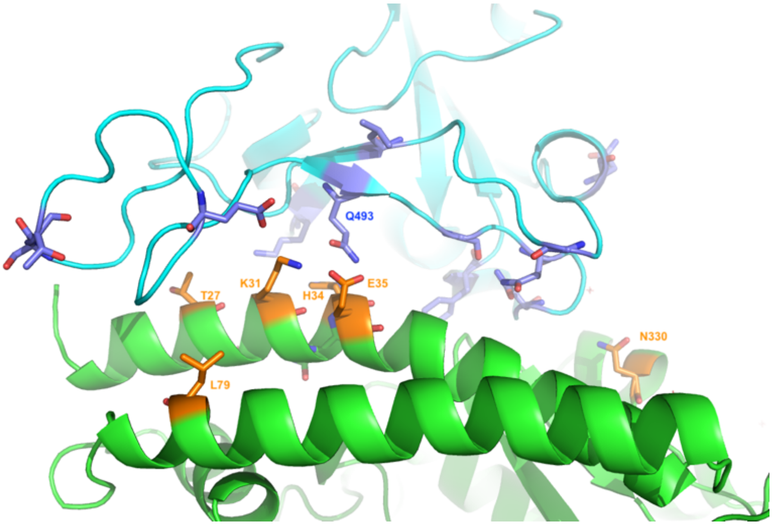

Fig. S4. ACE2 and RBD complex structure. Mutations observed in the spike proteins of different variants of concern are shown as sticks in blue. Mutations identified in the ACE2 peptidase domain that could potentially enhance RBD binding are shown as sticks in orange. T27, L79, N330 residues in ACE2 are all too far away (beyond 10 Å) from mutation positions observed in SARS-CoV-2 VOCs, thus mutations of T27, L79, N330 residues in ACE2 should not impact ACE2 binding affinity to different SARS-CoV-2 VOC RBDs. H31, H34, E35 residues in ACE2 are relatively close to Q493R

mutation in the Omicron variant. For H31F, H34A or H34I mutations in ACE2, they don't seem to have direct interactions with Q493 in RBD, thus H31F, H34A or H34I mutations in ACE2 probably will not impact ACE2 binding affinity to different SARS-CoV-2 VOC RBDs. E35Q in ACE2 may have hydrogen bond interaction with Q493 in wild type RBD, this hydrogen bond interaction could also be retained in Q493R mutation in the Omicron variant. Given this is a solvent exposed hydrogen bond interaction, the free energy contribution to binding of the hydrogen bond will be minimal, thus the hydrogen bond interaction probably will not enhance or disrupt binding affinity between ACE2 and RBD in different VOCs.

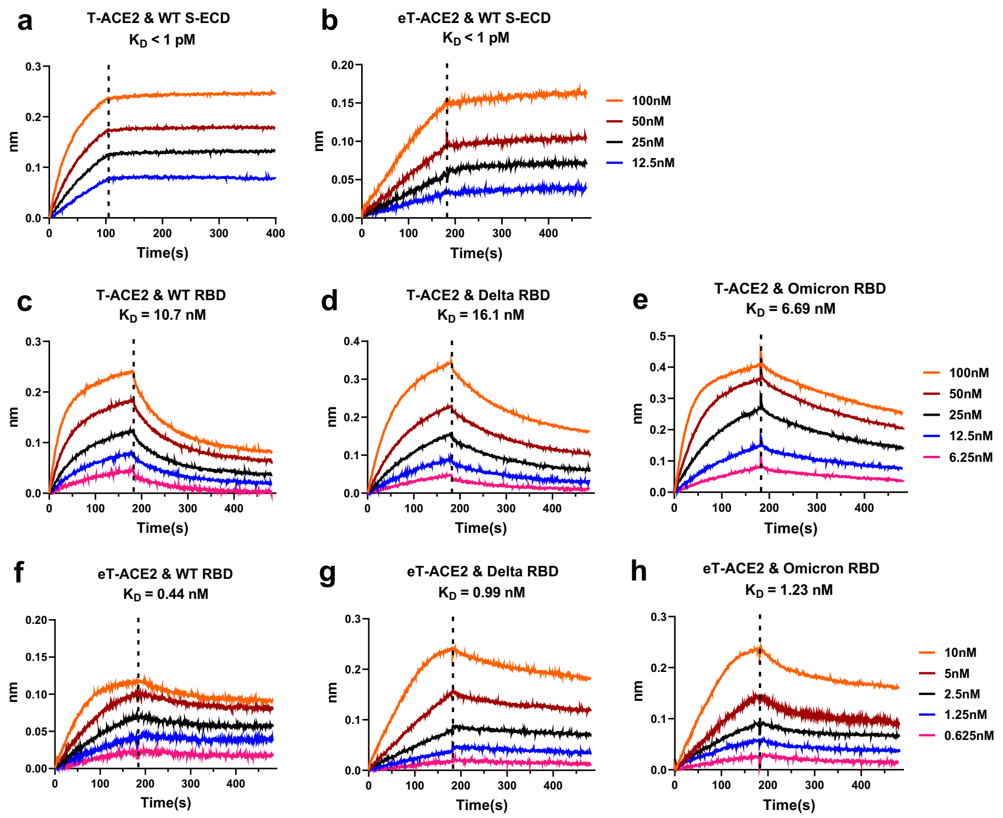

Fig. S5. Binding affinity measurement using biolayer interferometry (BLI). a. T-ACE2 binding with wild type (WT) spike protein (S) ECD. **b.** eT-ACE2 binding with wild type spike protein ECD. **c.** T-ACE2 binding with wild type RBD. **d.** T-ACE2 binding with Delta variant RBD; **e.** T-ACE2 binding with Omicron variant RBD. **f.** eT-ACE2 binding with wild type RBD. **g.** eT-ACE2 binding with Delta variant RBD. **h.** eT-ACE2 binding with Omicron variant RBD.

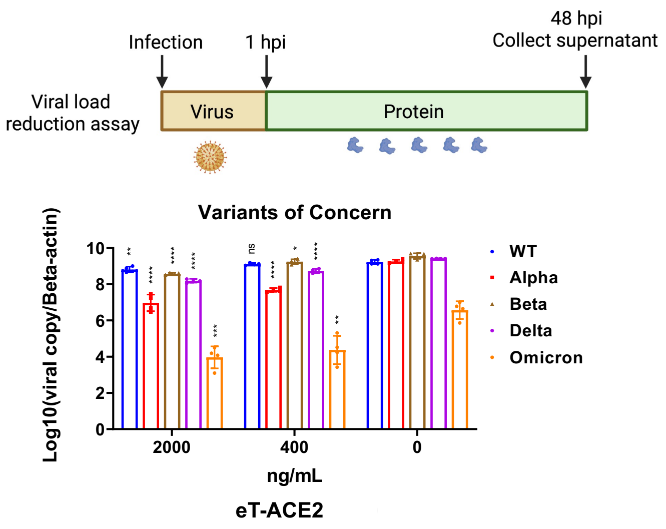

Fig. S6. eT-ACE2 inhibition of different authentic SARS-CoV-2 variants of concern in a viral load reduction assay. eT-ACE2 groups' P value were analyzed by ordinary one-way ANOVA test, bar graphs represent mean $\pm$ S.D., ns. $P > 0.05$, * $P \leqslant 0.05$, ** $P \leqslant 0.01$, *** $P \leqslant 0.001$, **** $P \leqslant 0.0001$.

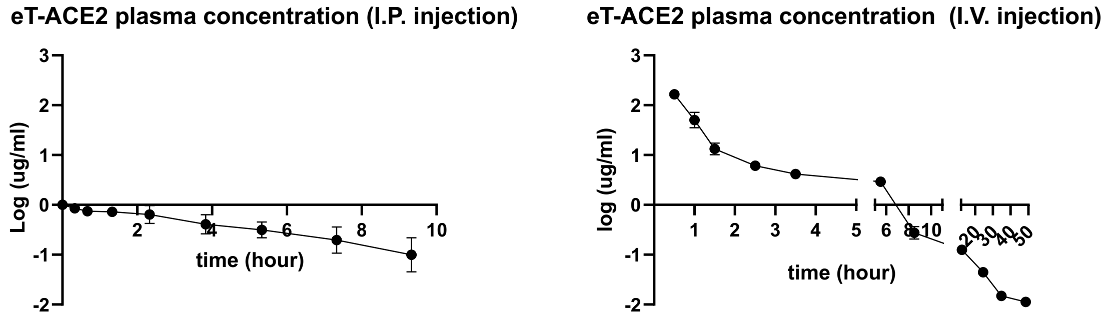

Fig. S7. Plasma concentration determination of eT-ACE2 in mice. eT-ACE2 was administered into mice through intraperitoneal (I.P.) injection or intravenous (I.V.) injection, blood samples were collected at different time points, eT-ACE2 concentration in each blood sample was then measured using sandwich ELISA.

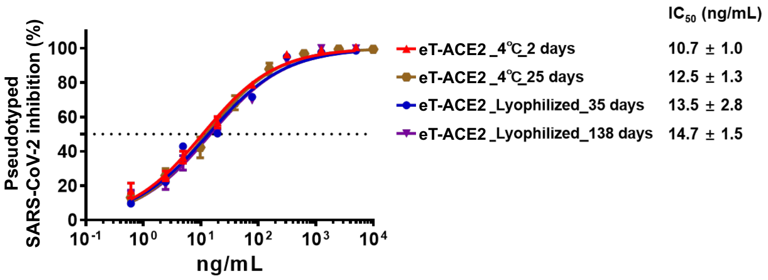

Fig. S8. eT-ACE2 activity can be maintained using different storage methods. eT-ACE2 protein was stored at 4 °C in PBS buffer for up to 25 days and the inhibitory activity was measured using wild type SARS-CoV-2 pseudovirus inhibition assay. eT-ACE2 was lyophilized and stored at -80 °C for up to 138 days, the lyophilized powder was then dissolved and the inhibitory activity was measured using wild type SARS-CoV-2 pseudovirus inhibition assay.

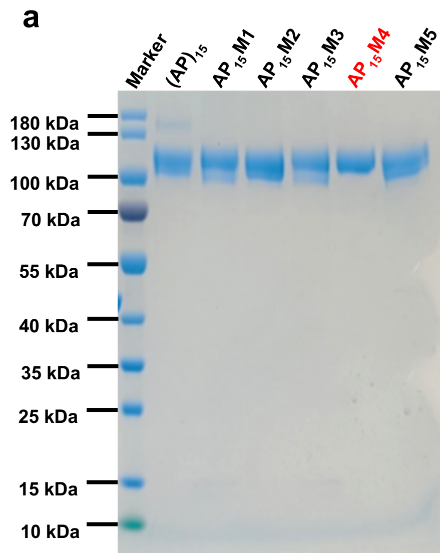

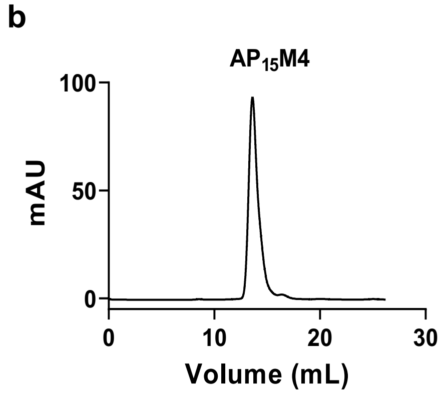

Fig. S9. Purified optimized trimeric ACE2 proteins. a. Different trimeric ACE2 proteins with $(AP)_{15}$ linker were prepared and the purity of these proteins were analyzed using SDS–PAGE with coomassie blue staining. **b.** The homogeneity of $AP_{15}M4$ protein was analyzed by size-exclusion chromatography.

References

1 Guo, L. *et al. Cell Res.* **31**, 98-100 (2021).

2 Lu, L. *et al. Clin. Infect. Dis.*, doi:10.1093/cid/ciab1041 (2021).

3 Yuan, S. *et al. Sci Adv* **6**, eaba7910 (2020).

4 Yuan, S. F. *et al. Nat Microbiol* **5**, 1439-1448 (2020).

5 Yuan, S. F. *et al. Nature* **593**, 418-423 (2021).

6 Glasgow, A. *et al. Proc. Natl. Acad. Sci. USA* **117**, 28046-28055 (2020).

7 Chan, K. K. *et al. Science* **369**, 1261-1265 (2020).

8 Tanaka, S. *et al. Sci. Rep.* **11**, 12740 (2021).
